# A Primer on Usability Assessment Approaches for Health-Related Applications of Virtual Reality

**DOI:** 10.2196/18153

**Published:** 2020-10-28

**Authors:** Timothy Zhang, Richard Booth, Royce Jean-Louis, Ryan Chan, Anthony Yeung, David Gratzer, Gillian Strudwick

**Affiliations:** 1 Centre for Addiction and Mental Health Toronto, ON Canada; 2 Faculty of Medicine University of Toronto Toronto, ON Canada; 3 Arthur Labatt Family School of Nursing Western University London, ON Canada; 4 Institute of Health Policy, Management and Evaluation University of Toronto Toronto, ON Canada; 5 Department of Psychiatry University of Toronto Toronto, ON Canada

**Keywords:** virtual reality, simulated environment, usability, evaluation, assessment methods, medical informatics, nursing informatics

## Abstract

Health-related virtual reality (VR) applications for patient treatment, rehabilitation, and medical professional training are on the rise. However, there is little guidance on how to select and perform usability evaluations for VR health interventions compared to the supports that exist for other digital health technologies. The purpose of this viewpoint paper is to present an introductory summary of various usability testing approaches or methods that can be used for VR applications. Along with an overview of each, a list of resources is provided for readers to obtain additionally relevant information. Six categories of VR usability evaluations are described using a previously developed classification taxonomy specific to VR environments: (1) cognitive or task walkthrough, (2) graphical evaluation, (3) post hoc questionnaires or interviews, (4) physical performance evaluation, (5) user interface evaluation, and (6) heuristic evaluation. Given the growth of VR in health care, rigorous evaluation and usability testing is crucial in the development and implementation of novel VR interventions. The approaches outlined in this paper provide a starting point for conducting usability assessments for health-related VR applications; however, there is a need to also move beyond these to adopt those from the gaming industry, where assessments for both usability and user experience are routinely conducted.

## Introduction

In the last decade, there has been a tremendous increase in the use of virtual reality (VR) technology in a variety of global contexts, including entertainment (eg, gaming), education, marketing, and design. VR broadly describes digitally created simulations where a person can be immersed in a computer-generated reality and complete tasks or interact with a virtual environment. Equipment such as VR headsets that allow individuals to experience the sounds and sights of a virtual world are often utilized to create an immersive experience.

More recently, numerous applications of VR specific to the health context have been identified and used [1-7], as research involving VR for health-related applications is gaining interest. As of July 2020, over 1000 studies were registered on ClinicalTrials.gov—a registry of clinical trials in the United States—for assessing VR interventions, such as anxiety management, distraction during painful procedures, gait training, rehabilitation, phobias, and medical education [8]. VR has been shown to be able to act as a low-cost and effective analgesic for pain arising in cases such as invasive medical procedures or even cancer in pediatric patients [9-11]. Hospitals may be able to leverage VR to reduce preoperative anxiety in patients, as well as a treatment method for those with generalized anxiety disorder [12]. A recent study by Donker et al showed that patients with acrophobia who received exposure therapy through a gamified, VR-enabled, self-help app had significant reductions in acrophobic symptoms [13]. Notably, in addition to the lack of need for a psychiatrist to be directly present during this intervention, the total cost per patient came to approximately US $24 through the use of Google Cardboard as the VR headset [13], exemplifying the ability of VR to increase treatment access while also significantly reducing costs. These examples only scratch the surface of the exciting potential of VR in health care.

To complement the significant amount of benefits that VR applications bring to health-related contexts, a focus on the usability of health information technologies needs to be maintained, particularly given the diverse needs and abilities of the user base (eg, patients, health professionals, family members, etc). By usability, we refer to how easily the technology can be utilized by an individual based on three cycles or steps [14]. Often, the effort invested into ensuring the usability of a technology or application goes unnoticed until the user interacts with a poorly designed system. A user’s proficiency with a technology may originate from a combination of their own self-exploratory learning as well as more formal, structured lessons and walkthroughs. Given the novel nature of VR in health care, the likely paucity of the latter places a greater emphasis on ensuring VR technologies are intuitive and easy to adopt for those who are new to the technology.

In the context of VR specifically, this includes both the use of the hardware (eg, headset) as well as the immersive software and VR experience as perceived by the user.

## Purpose

The purpose of this viewpoint paper is to conduct the following: (1) highlight the need to conduct usability assessments for VR apps, (2) provide a primer on the potential usability assessment approaches that can be applied to VR in health-related contexts and their potential challenges, and (3) direct readers to several resources where additional information on the topic can be found.

## The Need to Conduct Usability Assessments for VR Health-Related Applications

One of the challenges of VR for health-related applications is assessing and addressing issues related to usability. Health-related applications of VR may warrant an even greater focus on usability testing than nonhealth-related applications, given that the user base (ie, those typically with illnesses, chronic conditions, or disabilities) is diverse in terms of ages, abilities, and beyond, and may have special needs that need to be accounted for when utilizing the technology. In addition, one of the most common problems associated with VR is motion sickness, which is often related to the quality of the virtual space mapping to the replicated physical setting [15]. This can be a significant barrier to users looking to obtain health-related benefits from using VR. Yet, there are methods in which motion sickness may be evaluated and addressed before the technology is implemented. The integration of VR into treatment plans can also meet commonly seen elements of friction associated with new technologies, such as distrust during adoption, although in some situations these can dwindle following introductory exposure [16]. Other limitations of contemporary VR include the challenge of generating varied types of tactile sensations [17] and other types of multisensory integration [18].

Assessment approaches for analyzing and evaluating the usability of various VR technologies for health-related applications have generally been understudied and not well described in the research literature. We conducted a cursory search ourselves of several academic databases and found limited explanations of usability methods utilized in the development stage of VR applications and an even more limited body of literature on how to conduct usability assessments for VR used for health-related purposes. While reasons for this gap in knowledge are likely due to the nascent nature of the field, further work must be completed toward generating best practices related to VR usability to assist practitioners and researchers in the development and diffusion of these sorts of innovations. For instance, outside of the VR context, there is an extensive literature base identifying the need for technologies that are used for health-related applications to be *user friendly* and have a high degree of *ease of use*, often incorporating in lessons from the human factors discipline [19-22]. Numerous papers, including one reporting on the System Usability Scale [23], have been published describing ways to assess usability for non-VR technologies, including electronic health records and mobile health apps [24-26]. Yet, there is limited guidance for those developing or researching health-related VR environments. Often, usability evaluation approaches used for other health information technology applications are difficult to implement within VR contexts. Thus, VR applications used in health contexts may not always undergo a thorough usability assessment. In the meantime, however, methods developed outside of the VR context will continue to be used until the scientific approaches for assessing VR usability further develop and until methods from the VR gaming industry become commonplace in health technology–related research.

## VR Usability Assessment Methods

### Overview

The following section describes VR usability assessment methods that have been employed in past research. It is important to note that these methods may be hybridized and blended together to suit the goals of each unique evaluation and are not mutually exclusive. The approaches are described using a previously developed classification of usability methods in virtual environments developed by Bowman and colleagues in 2000 [27] and updated by Martens in 2016 [28]. These approaches include (1) cognitive or task walkthrough, (2) graphical evaluation, (3) post hoc questionnaire or interview, (4) physical performance evaluation, (5) user interface (UI) evaluation, and (6) heuristic evaluation.

Table 1 [14,21,29-36] summarizes key information related to each of the identified VR assessment approaches, including some considerations for assessment requirements excluding basic needs, such as an appropriate space to conduct a VR assessment and the VR hardware and software itself.

It is recommended that some assessment methods should favor the involvement of specific user groups, such as external users (ie, a group of testers not involved in the development process). Some assessment method requirements also lend themselves to requiring representative users, meaning a sample of users who may reflect the appropriate end-user population. The following sections provide an explanation of each of the VR usability assessment approaches.

**Table 1:** Overview of virtual reality (VR) usability assessment approaches.

Approach	Assessment requirements^a^	VR aspects evaluated	Typical output of assessment	Results type
Cognitive or task walkthrough [14,29]	Representative external users, developed task or scenario, and recording and timing equipment	Environment navigation, object interaction, and user-system interaction	Task performance and user feedback	Discrete and descriptive
Graphical evaluation [30,31]	Multiple relevant graphical environments, *recording equipment, questionnaires,* and *interview guides*	Quality of graphics and image renderings	User feedback	Descriptive
Post hoc questionnaires and interviews [32]	External users, questionnaires or interview guides, and *recording equipment*	Nonspecific	User feedback	Descriptive
Physical performance evaluation [33]	External users, developed task or scenario, and *recording and timing equipment*	Physical immersion and VR performance	Task performance, system performance metrics, and user feedback	Discrete and descriptive
User interface evaluation [14,34,35]	Developed task or scenario, *recording and timing equipment, questionnaires,* and *interview guides*	Integration of VR environment and real-life tools and VR performance	User feedback and task performance	Discrete and descriptive
Heuristic evaluation [14,21,36]	Experienced users, developed task or scenario, questionnaires, interview guides, and *recording equipment*	Various	Refer to list in Heuristic Evaluation section	Descriptive

^a^Italicized text indicates optional requirements depending on specific assessment approaches being used (eg, recording equipment is only required if incorporating think-aloud methods).

### Cognitive or Task Walkthrough

The cognitive or task walkthrough is a formative assessment method that assesses the user, or hypothetical user, based on the completion of task-based VR scenarios, response to system changes, and the user’s exploration and navigation of the VR environment [14]. While other measures for task load performance exist, such as the NASA-TLX (Task Load Index) [37], this assessment is based on Norman's 1986 [38] model of interaction and assesses the user’s mental and physical actions in VR environments founded on the premise that users learn to use a technology through a process of self-exploration rather than didactic training or lessons [39]. Originally designed to assess simple UIs, such as automated teller machines and kiosks, this assessment method is increasingly used to assess VR usability as well [40].

One way to perform such an assessment is by employing the following three cycles or steps. The first cycle assesses a user’s actions when they are trying to achieve a goal [14]. An observer will document the overall path the user takes to complete a task or whether they behave in an *intended* way in the VR scenario. Challenges or issues in achieving the goal of each cycle are noted by the observer. Behaviors in this first cycle are largely dictated by the user having to make decisions and how the environment facilitates such decision pathways. For example, if the user’s goal is to pick up an object, but the object is missing, then the environment should allow the user to locate the object. Locating the object itself leads to the second cycle or step called “exploration and navigation in virtual environments” [14].

In the second cycle, the user explores and moves around the environment to identify a path toward an object of interest. The VR environment should allow for intuitive navigation, recognizing user movements and responsively adapting to changes in user location as the user explores to locate the object of interest [14]. The observer records any challenges or issues in achieving this goal.

In the third cycle, the user’s behaviors in response to a system initiative are assessed [14]. The purpose of this cycle or step is to examine how the VR system supports user activity when the user manipulates an object. The user and system are required to reciprocally recognize and interpret the feedback or actions of one another and respond appropriately [14]. For instance, if the user decides to throw a vase, the system should interpret this action and produce an appropriate response, such as depicting the vase flying and being shattered when contacting another object such as a wall. Correspondingly, the system may also take the initiative and act, meaning it is the user’s role to interpret and respond to this action [14]. For example, if a helium balloon (ie, the object) suddenly detaches from its base and starts floating away (ie, the system’s action or initiative), the user in this case may then choose to intervene and attempt to catch the balloon or allow the balloon to float away.

In summary, a cognitive or task walkthrough is a task-based assessment that assesses a user’s actions when they are trying to achieve a goal (see Table 1) and incorporates further assessments of user navigation (ie, cycle two) and system response (ie, cycle three). For each of these cycles, users should be allowed to freely walk through the task or interaction without interruption by the observers. Since this approach is largely driven by scripts and dialogues within the VR environment, usability issues and the system’s ability to support user interaction is primarily assessed through descriptive, qualitative feedback (eg, user comments, think-aloud method, and observer observations) [14,28].

### Graphical Evaluation

This assessment method focuses on the quality of graphics generated in the VR environment and how it influences the user's experience. This may include, but is not limited to, how different color combinations, shapes, textures, and renderings depicted will impact the user’s interaction with the VR environment and system [30,41]. There are numerous ways to assess graphics, which can be attuned to hardware (eg, view, resolution, color contrast, update rate, etc); fidelity (eg, geometry and colors); camera placement, if applicable; the precision of the tracking system; stereoscopic image quality [42]; and beyond [43].

Many methods that vary in degrees of complexity exist for assessing graphics. In one common approach, users are exposed to different iterations of graphical environments to get a better understanding of its impact on user experience [30,41]. Depending on the purpose of the overall VR environment, the graphical object of interest may vary. For example, to examine a user’s behavior in a large city, the graphical evaluation may be more focused on image depth, complexity, and breadth of the city and its 3D renderings. If the focus is narrower, such as assessing how a user reacts to seeing smoking paraphernalia, then focusing on meticulous, realistic details for an object such as a cigarette will be of greater importance. To assess a user’s response to graphics in a VR environment, users may be asked to think aloud or be given a set of questionnaires to collect user feedback about the graphical output in the VR system (see Post Hoc Questionnaires and Interviews section) [30,41].

### Post Hoc Questionnaires and Interviews

Post hoc questionnaires and interviews are often used to identify a user’s general overall experience in using VR. However, some of these questionnaires and interviews may be targeted toward specific usability concepts, such as graphics, the physical hardware, and motion sickness [37,44,45].

This assessment method is often performed following the conclusion of a user’s interaction with the VR system [28]. Since VR remains a relatively new technology, responses may be highly influenced by the individual’s comfort and experience with using VR. Thus, unless the usability evaluation is already tailored to a target or only includes a subset of users based on experience (eg, inexperienced VR users), demographic information about users’ opinions, views, and experiences with VR should also be collected to help better interpret user feedback [28]. Due to its versatility, this assessment can be viewed as a complement to many of the assessments covered in this article rather than a stand-alone method. Its overall purpose is to serve as a simple, straightforward way of collecting targeted feedback. In order to collect specific feedback pertaining to the specific evaluation tied to a post hoc questionnaire or interview, special care must be given to the semantics and framing of questions [28].

Often, post hoc questionnaires are also used during the VR prototyping stage by engaging end users as a form of iterative quality improvement, but often in conjunction with another evaluation method such as a cognitive or task walkthrough [28].

### Physical Performance Evaluation

Physical performance in the context of VR is defined by the performance of the hardware and environment. The smoothness and quality of the virtual environment are evaluated not unlike how a website can be evaluated on its loading time. Performance metrics with this assessment method include lag time (ie, the time delay between the user’s intended action and the system’s response within VR) and synchronization (ie, whether the system accurately reflects the user’s intended actions). VR should be as convincingly realistic as possible to users, and the physical performance of a VR system is the key determinant of mental and physical immersion [33]. Immersion is defined as a state of being fully absorbed and/or deeply engaged within a simulated environment and is a key factor in determining the quality of VR [21]. This assessment method can facilitate user-centered design and can also yield information on the physical space required for users to fully explore the VR environment [33].

To gauge the VR system’s physical performance, data can be obtained through a combination of approaches, such as questionnaires, task performance scores, or by leveraging back-end data to examine factors such as retrieval and load times. Simple but physically demanding VR precision tasks are highly informative for this type of assessment. For example, a task involving manipulating small objects with virtual chopsticks will quickly reveal any performance issues related to the precision of translated movements. Such a task can be timed and scored, and the user can be asked to describe their satisfaction and feelings to identify physical performance issues [33]. As another example, tasks involving actions that require users to reach out around their *body* to interact with nearby objects can be used to highlight unaddressed issues with distance compression, a frequent phenomenon within VR environments where objects are perceived by the user to be closer than their actual position [46]. Following a given task, a user may achieve high task performance scores but still report heavy cognitive overload (ie, mental exhaustion) while using the system, for example, finding that performing the task in VR was significantly more difficult than performing the same task with real objects or tools. Such a situation would signal that some probing questions (eg, Was there a specific action of the task that was particularly difficult to perform?) or further back-end evaluations may be required to identify possible underlying physical performance–related issues [33].

### User Interface Evaluation

The purpose of a UI evaluation is to help determine the usability of a VR system’s front-end UI [14,35,47]. This approach can also help identify a UI design or solution that appropriately balances factors such as intuition and immersion against usability [34]. An optimized UI solution should provide the user with the best combination between immersion and usability, such that users feel immersed but unencumbered in accomplishing their tasks relative to outside a VR environment [14,34]. A feeling of immersion is especially pertinent when considering VR applications that notably outperform real-world counterparts, such as a simulated environment used to manage phobias or pain [8]. In these unique situations where the UI itself is deeply interrelated with the intervention (ie, phobia exposure tool), a comprehensive UI design evaluation may only be feasibly accomplished by a wider-scale clinical trial measuring treatment outcomes. Returning to more general VR applications, a UI evaluation allows for the identification of the type of UI solution that will provide the best immersion-to-efficiency ratio between a VR environment and real-life tools [31]. In a proof-of-concept case study by Kasurinen [34], users were instructed to complete one of five training scenarios with three varying levels of VR and real-life tools [34]:

1. No VR: participants move throughout an environment with keyboard and mouse controls; other activities are completed with real-life tools in a simulated workspace setting.
2. Semi-VR: participants move within a virtual environment with a VR headset; other activities are completed with real-life tools in a simulated workspace setting. The real-life workstation also displays the current state of the VR.
3. Full VR: participants move and complete their activities fully within a VR environment. Real-life tools are replaced with virtual equivalents (eg, virtual keyboard) and other real-life displays (eg, workstation screen) are virtually broadcasted to the VR headset.

For each iteration, data on user preferences can be collected alongside discrete data, such as task completion times and the number of errors [34]. Questions related to UI elements should also be asked throughout each iteration, as follows [14]:

1. Can the user form or remember the task goal?
2. Are the appropriate objects or parts of the environment viable?
3. Can the necessary objects be located?
4. Can the user execute movement and navigation actions?
5. Can the user recognize objects in the environment?

Each of these questions can help to reveal a specific area with potential for improvement within the UI. This method can aid in assessing both the appropriate amount of real-life integration and the quality of said integration so the VR intervention can best accomplish its intended purpose. If the integration between virtual and real-life tools is insufficient, it has been shown that this friction will cause users to prefer the *No VR* option, which may also be partially related to physical performance (see Physical Performance Evaluation section) [34].

### Heuristic Evaluation

A heuristic evaluation is a UI approach that involves several topic experts or an expert evaluator, rather than soliciting direct user feedback. A VR usability expert will typically evaluate a UI’s design against an accepted set of usability principles or standards already published in the literature [48]. While there are several sets of accepted standards or heuristics, for traditional UIs little research exists on defining heuristics for VR environments. Nielsen’s [21] heuristics set is the most commonly referenced and utilized set of heuristics for UI design. Sutcliffe and Gault [48] further defined a set of 12 heuristic guidelines based on Nielsen’s set, as shown in Textbox 1.

**A set of 12 heuristic guidelines.**

- Natural engagement
- Compatibility with the user’s task and domain
- Natural expression of action
- Close coordination of action and representation
- Realistic feedback
- Faithful viewpoints
- Navigation and orientation support
- Clear entry and exit points
- Consistent departures
- Support for learning
- Clear turn taking
- Sense of presence

Expert results are then aggregated and used to identify priority areas of action [28]. Heuristic assessments also require a set of tasks for the experts to experience. The nature of these tasks and the VR environments themselves should also be subjectively considered when carrying out a heuristic assessment, given the lack of standardization between various types of VR equipment and software [28]. While not all heuristics may apply to a given VR application, such an evaluation has great potential to glean a rich, overall picture of the state of the application. For example, if the VR application is intended to be designed in a way that the user is automatically placed in an “inescapable” environment, then there is no relevance in assessing clear entry and exit points (ie, the eighth heuristic guideline, *clear entry and exit points*) [28]. Since heuristics are broad rules of thumb rather than specific guidelines, they should not be treated as binary checkboxes, but rather as individual continuums that can each be an area for improvement, although binary elements may exist within. To illustrate, perhaps the heuristic guideline of *realistic feedback* is of particular interest, which outlines that the VR application should help users effectively recognize and recover from errors [21]. The presence or absence of a feature such as, for example, tangible error messages would constitute a binary checkbox, but the palatability and effectiveness of said error messages would be of higher importance. Is the problem or error precisely and concisely indicated? Is a potential solution suggested? Is the language user friendly and free of codes or abbreviations, such as “A 50 (0x32) error has occurred”? Ultimately, considering and tracking multiple granular elements within each heuristic will aid greatly in obtaining actionable results to direct improvement.

## Resources Where Additional Information on the Topic Can Be Found

Table 2 [14,21,23,29-34,36,37,39,40,42-45,49-54] provides a list of references specific to each of the approaches where readers can access additional information.

**Table 2:** Additional resources.

Assessment approach	References and resources
Cognitive or task walkthrough	[14,29,39,40,49,50]
Graphical evaluation	[30,31,42,43]
Post hoc questionnaires and interviews^a^	[23,32,37,44,45,51]
Physical performance evaluation	[33]
User interface evaluation	[14,34]
Heuristic evaluation	[14,21,36,52]
Other	[53,54]

^a^This represents a sample of many that can be employed, depending on what usability concept is to be measured.

## Other Considerations

As previously noted, many of the approaches presented in this paper can be blended or hybridized together to suit the goals or needs of a given VR application evaluation. They are certainly not mutually exclusive. Some of the methods already incorporate a level of hybridization, most often with the inclusion of a post hoc questionnaire or interview. Given the lack of standardization across approaches, this warrants future research regarding the development of a comprehensive framework incorporating multiple methods of VR evaluation to provide, at a minimum, a strategic work plan for those looking to perform a baseline evaluation of any new VR application. This should include the incorporation of more up-to-date methods already used in the gaming industry. Those who employ usability methods for VR that have been developed for other kinds of health information technologies should be encouraged to share their experiences with the broader scientific community, placing an emphasis on the practical experiences of doing so. The current literature base lacks practical examples of how to best use these approaches, which could be of great use to those employing them.

When developing VR interventions and applications, particularly in the context of health, the comfort of the end user is paramount. Alongside the numerous benefits of VR technology, VR still carries the risk of imposing symptoms similar to motion sickness during use as a result of visual distortions and asynchronies, among other effects [45]. While these issues are peripherally related to performance issues and may be identified in user feedback, these data are inherently subjective and the effects are, thus, not easily quantifiable enough to measure improvements. Thus, the authors recommend that any VR assessment also explicitly consider the possible effect of motion sickness on its users by incorporating tools such as the Simulator Sickness Questionnaire (SSQ), originally developed to help measure motion sickness for pilots in flight simulators [45]. The results from the SSQ or another similar questionnaire may identify specific considerations for certain populations, age groups, diagnoses, and beyond. Additionally, the repeated occurrence of specific symptoms or combinations of such from the SSQ (eg, eyestrain, nausea, and vertigo) can provide additional direction in identifying the root issues within the VR software and hardware [45].

## Conclusions

Health-related applications using VR are a rapidly advancing area of development. Like all emerging technologies in health care, there is a need to ensure the quality and safety of these novel tools [55]. For VR, validated usability and assessment approaches are an important step before its deployment in real-world clinical settings. The assessment methods described here give developers and researchers a high-level overview of important elements to consider regarding the usability of their VR implementations and to make iterative changes prior to clinical implementation. However, once these approaches are employed for VR, sharing practical experiences in doing so would be of tremendous value. This area of science is in its infancy and comprehensive knowledge translation would be critical to its growth.

Overall, this paper provides a description and discussion of six different contemporary VR usability assessment methods. As an emerging area for research, the development of formative usability assessment methodologies for health-related VR applications is an important area for future development. Further, while the six approaches discussed in this paper have been discussed in isolation, further future hybridization of approaches to develop more robust and multidimensional interpretations of VR usability should be considered. For instance, like other usability evaluation approaches [21,56], a purposeful mixed methods approach may assist in generating more holistic and robust interpretations of a system’s usability. We see value in the triangulation of data related to user feedback and other task performance metrics in health-related VR applications. Due to the nascent nature of the domain, a pluralistic approach to usability evaluation should be considered in an effort to develop broader and more nuanced understandings of the state of the art in VR.

Given that the VR industry is projected to grow to over US $9 billion in sales of VR devices alone by 2021 [57], it is no surprise the industry is marked with large financial investments, such as the acquisition of Oculus for US $2 billion, as many large technology companies continue to invest heavily in VR [58]. As a collective, health care organizations and professionals should emphasize ensuring the mitigation and prevention of potential growing pains that may arise if VR interventions are churned out without rigorous evaluation and proper regard for quality, allowing for VR to usher in a new field of innovative, technology-enabled health care. With this foundation, the potential benefits to providers and patients alike will only continue to grow with continuous improvements in technology and reductions in cost.

## Abbreviations

ICMJE: International Committee of Medical Journal Editors
SSQ: Simulator Sickness Questionnaire
TLX: Task Load Index
UI: user interface
VR: virtual reality
